# Influence of Lithium- and Zinc-Containing Bioactive Glasses on Pulpal Regeneration

**DOI:** 10.1055/s-0042-1758789

**Published:** 2023-02-22

**Authors:** An Thi Loc Tran, Charnsak Sukajintanakarn, Pisol Senawongse, Hathaitip Sritanaudomchai, Nisarat Ruangsawasdi, Puangwan Lapthanasupkul, Nakarin Kitkumthorn, Naruporn Monmaturapoj, Chutikarn Khamsut, Parichart Naruphontjirakul, Pong Pongprueksa

**Affiliations:** 1 Dental Biomaterials Science Program, Faculty of Dentistry, Mahidol University, Bangkok, Thailand; 2 Department of Conservative Dentistry and Prosthodontics, Faculty of Dentistry, Srinakharinwirot University, Bangkok, Thailand; 3 Department of Operative Dentistry and Endodontics, Faculty of Dentistry, Mahidol University, Bangkok, Thailand; 4 Department of Oral Biology, Faculty of Dentistry, Mahidol University, Bangkok, Thailand; 5 Department of Pharmacology, Faculty of Dentistry, Mahidol University, Bangkok, Thailand; 6 Department of Oral and Maxillofacial Pathology, Faculty of Dentistry, Mahidol University, Bangkok, Thailand; 7 Assistive Technology and Medical Devices Research Center, National Science and Technology Development Agency, Pathum Thani, Thailand; 8 Biological Engineering Program, Faculty of Engineering, King Mongkut's University of Technology Thonburi, Bangkok, Thailand

**Keywords:** bioactive glass, lithium bioactive glass, zinc bioactive glass, tooth culture model, fibrinogen thrombin

## Abstract

**Objective** To evaluate the potential of modified bioactive glasses containing lithium and zinc as pulp capping materials by investigating the odontogenic differentiation and mineralization response in the tooth culture model.

**Materials and Methods** Lithium- and zinc-containing bioactive glasses (45S5.1Li, 45S5.5Li, 45S5.1Zn, 45S5.5Zn, 45S5.1Zn sol-gel, and 45S5.5Zn sol-gel), fibrinogen-thrombin, and biodentine were prepared to assess *Axin2* gene expression at 0, 30 minutes, 1 hour, 12 hours, and 1 day and *DSPP* gene expression at 0, 3, 7, and 14 days in stem cells from human exfoliated deciduous teeth (SHEDs) using qRT-PCR. The experimental bioactive glasses incorporated with fibrinogen-thrombin and biodentine were placed on the pulpal tissue in the tooth culture model. Histology and immunohistochemistry were analyzed at 2 weeks and 4 weeks.

**Results** *Axin2* gene expression for all experimental groups was significantly higher than the control at 12 hours. The *DSPP* gene expression for all experimental groups was significantly higher than the control at 14 days. The presence of mineralization foci was significantly higher at 4 weeks for the modified bioactive glasses 45S5.5Zn, 45S5.1Zn sol-gel, and 45S5.5Zn sol-gel as well as Biodentine compared with the fibrinogen-thrombin control.

**Conclusion** Lithium *-* and zinc *-* containing bioactive glasses increased *Axin2* and *DSPP* gene expression in SHEDs and can potentially enhance pulp mineralization and regeneration. Zinc *-* containing bioactive glasses are a promising candidate to be used as pulp capping materials.

## Introduction

Vital pulp therapy is a treatment modality to preserve and maintain the health of pulpal tissue in teeth that have been exposed to trauma, dental caries, or restorative procedures. The purpose of vital pulp therapy is to promote tertiary reparative dentin or dentin bridge formation. Maintenance of pulp vitality can reduce the risk of tooth fracture and aid in continued tooth development as the integrity of the natural tooth structure is conserved. 1 To achieve this, direct pulp capping materials that contact the pulpal exposure site are used.

Biodentine, a form of tricalcium silicate (Ca _3_ SiO _5_ ), is available as a powder composed of tricalcium silicate, dicalcium silicate, calcium carbonate, zirconium oxide, and iron oxide, which is mixed and activated with the liquid component that contains calcium chloride as an accelerator, giving a setting time within 12 minutes. 2 The advantages of Biodentine as a pulp capping material are its biocompatibility, good antimicrobial activity, 3 ability to stimulate tertiary dentin formation, 4 has mechanical strength comparable to dentine, and low solubility. 5 An *ex-vivo* study that evaluated the use of Biodentine in a tooth culture model found mineralized foci formation soon after its application and also significantly increased TGF- β1 secretion from pulpal cells. 6

Bioactive glasses have widely been used in medical applications, such as bone grafts, tissue scaffolds, coating materials, and for treatment of hypersensitivity. 7 Their highly desirable properties include excellent biocompatibility, bioactivity, 7 8 osteoinduction, and osteoconduction. 7 8 9 In dentistry, these materials have increasingly been of interest to promote dental pulp differentiation and mineralization. 10 11

The Wnt/β-catenin signaling pathway plays a significant role in pulpal cell differentiation, and the formation of reparative dentine. 12 13 Activation of this pathway includes the inhibition of glycogen synthase kinase 3 (GSK-3), one of the protein kinases that degrades β-catenin. The deactivation of GSK-3 by GSK-3 inhibitors will lead to the up-regulation of the axis inhibition protein 2 ( *Axin2* ) gene, 12 14 which encodes for the expression of dentin sialophosphoprotein (DSPP) that plays an important role in the differentiation of mesenchymal cells to odontoblast like-cells and the subsequent mineralization of dentine matrix. 13 As lithium and zinc ions have been shown to be GSK-3 inhibitors, these materials are gaining attention in dental materials science to be incorporated into bioactive glasses used for pulp capping. 15 16

The *ex-vivo* tooth culture model was recently introduced to investigate the early stages of dentin regeneration using an entire human tooth, which is conditioned with pulp capping materials. 17 18 19 This model has been able to demonstrate cell migration, 20 the differentiation of progenitor cells into odontoblast-like cells, 17 21 and the early stages of reparative dentine formation. 6 Thus, the tooth culture model has been a valuable technique for investigating the biocompatibility, pulpal reaction, and mineralization effects of experimental dental pulp capping materials.

The objective of this study was to evaluate the influence of bioactive glasses containing lithium and zinc on the Wnt/β-catenin signaling pathway, particularly on *Axin-2* and *DSPP* gene expression, in stem cells from human exfoliated deciduous teeth (SHEDs). Another outcome assessed in this study was the effect of this composite biomaterial on odontogenic differentiation and mineralization at 2 and 4 weeks using the tooth culture model.

## Materials and Methods

The compositions of the modified bioactive glasses are presented in Table 1 . Modified bioactive glasses containing lithium (45S5.1Li, 45S5.5Li) and zinc (45S5.1Zn, 45S5.5Zn) were synthesized using a melt-quenching process, provided by the National Metal and Materials Technology Center. The required proportions of the raw materials were mixed together, melted in a covered Pt-10%Rh crucible at 1,450°C for 2 hours and then quenched in cold water to produce frit. Fine glass powder was produced by ball milling with a zirconia ball as the grinding media to achieve an average particle size (D0.5) of 5.81 μm upon being analyzed by laser diffraction technique (Mastersizer 2000, Malvern Instruments, Worcestershire, UK). Another two groups of Zn-containing modified bioactive glasses (45S5.1Zn sol-gel, 45S5.5Zn sol-gel) were synthesized using the sol–gel technique, provided by King Mongkut's University of Technology Thonburi. TEOS (tetraethyl orthosilicate 98%) and TEP (triethyl phosphate 99%) were added to nitric acid (0.1 M) and ethanol. The reagents were mixed for 1 hour using magnetic stirring at room temperature. Then, sodium nitrate (NaNO _2_ ) and calcium nitrate tetrahydrate (Ca(NO _3_ ) _2_ .4H _2_ O) were dissolved in the sol, and magnetic stirring was maintained for another hour. After that, zinc nitrate hexahydrate (Zn(NO _3_ ) _2_ 6H _2_ O) was added to the mixture according to the required proportions of the raw materials and mixed together. The obtained gel was placed in a sealed polytetrafluoroethylene (PTFE) large screw-top container and aged for 72 hours at 60°C before the container was opened and dried at 100°C for 48 hours. Samples were thermally treated at 550°C for 3 hours at a rate of 3°C/min. Finally, the samples were ground and sieved, and the particle fraction below 150 µm was evaluated. Fibrinogen and thrombin were purchased from Baxter (Tisseel kit: Baxter), and diluted with tris-buffered saline solution to obtain a final concentration of 49.42 mg/mL and 16 units/mL, respectively, after which the two solutions were mixed at a 1:1 ratio.

**Table 1:** Compositions of lithium- and zinc-containing bioactive glasses used in this study (wt%)

	SiO _2_	Na _2_ O	CaO	P _2_ O _5_	Other
45S5.1Li	45	23.5	24.5	6	1 Li _2_ O
45S5.5Li	45	19.5	24.5	6	5 Li _2_ O
45S5.1Zn	45	23.5	24.5	6	1 ZnO
45S5.5Zn	45	19.5	24.5	6	5 ZnO
45S5.1Zn sol-gel	45	23.5	24.5	6	1 ZnO
45S5.5Zn sol-gel	45	19.5	24.5	6	5 ZnO

### Cytotoxicity Test (MTT Assay)

*Material preparation* : In total, 0.2 g of each modified bioactive glass powder ( *n* = 3) was UV-sterilized for 15 minutes before being soaked separately in 1 mL of a medium supplement consisting of Dulbecco's modified Eagle medium (DMEM; Hyclone), 10% fetal bovine serum (FBS; Biochrom), and 1% penicillin/streptomycin (Gibco, Life Technologies) in a shaker (PSU-2T Mini-shaker, Biosan) at 37°C in 5% CO _2_ for 24 hours. The extracted medium was filtered using a membrane filter of 0.22 μm pore size (Millipore).

The mixture of Fibrinogen-Thrombin and Biodentine (Septodont) was placed into a Teflon mold measuring 5 mm in diameter and 2 mm in thickness and covered between two glass slides. All specimens were UV-sterilized on each side for 15 minutes. Four specimens were incubated in 1 mL of a medium supplement for each experimental group ( *n* = 3), and the extracted medium was filtered as above.

*MTT assay* : Stem cells from human exfoliated deciduous teeth (SHEDs) were isolated, cultured, and characterized as from a previous study. 22 The cells were cultured in a medium supplement medium consisting of DMEM with 10% FBS and 1% penicillin/streptomycin at 37°C in 5% CO _2_ . This procedure was performed for all experimental materials using the cells from passage 6 onward to 10.

The SHEDs were cultured in a 96-well plate (Nunc, Thermo Scientific) at a density of 10 ^4^ cells per well at 37°C in 5% CO _2_ for 24 hours. The cells were treated with a diluted extracted medium in triplicate at 2,500 μg/mL and 1,000 µg/mL concentrations of 45S5.1Li, 45S5.5Li, 45S5.1Zn, 45S5.5Zn, and a control, then incubated at 37°C for 24 hours. The medium was aspirated, and 50 µL of the MTT reagent of 1 mg/mL concentration was added. After incubation for 2 hours, the solution was removed and replaced with 100 µL of isopropanol (Ajax Finechem, Thermo Fisher Scientific). The culture plate was lightly shaken for 30 minutes on a microplate shaker. The optical density (OD) at 570 nm for each sample was then measured using a spectrophotometer (Epoch Microplate Spectrophotometer, BioTek).

### Axin2 and DSPP Gene Expression

*Material preparation* : The extracted medium with a 2,500 μg/mL concentration of experimental materials (45S5.1Li, 45S5.5Li, 45S5.1Zn, 45S5.5Zn, 45S5.1Zn sol-gel, 45S5.5Zn sol-gel) were evaluated ( *n* = 3). Four specimens of Biodentine mixture (0.2 g) were prepared in a Teflon mold (5 mm in diameter and 2 mm in thickness) and immersed in 1 mL of culture medium for 24 hours. The extracted medium was diluted to the same concentration of bioactive glass. Another four specimens of the Fibrinogen-Thrombin mixture were placed into a Teflon mold and processed to a similar dilution with Biodentine. The negative control medium comprised DMEM, 10% FBS, and 1% penicillin/streptomycin, while the positive control medium was an osteogenic differentiation medium (AdvanceSTEM) with 10% stem cell growth supplement (AdvanceSTEM) and 1% penicillin/streptomycin (Gibco, Life Technologies).

*Gene expression:* Axin2 and dentin sialophosphoprotein ( *DSPP* ) gene expression were determined using a quantitative real-time reverse transcription-polymerase chain reaction (qRT **-** PCR). The SHEDs were cultured in a 6-well plate (Nunc, Thermo Scientific) at a density of 1.5 × 10 ^5^ cells/well at 37°C in 5% CO _2_ for 24 hours. Once the extracted medium was added to the SHEDs, *Axin2* gene expression which represents the early process of odontoblast-like cell differentiation through the Wnt/β-catenin pathway was analyzed immediately (0 minutes), and after 30 minutes, 1 hour, 12 hours, and 1 day. DSPP RNA expression which represents modulation of dentin mineralization was evaluated immediately (Day 0), and after 3, 7, and 14 days, where the extracted medium was changed every 3 days at 2 mL/well. The RNA was first extracted using Trizol reagent (Invitrogen) before being treated with a DNase I, RNase-free kit (Thermo Fisher Scientific) to remove genomic DNA from the RNA preparations. In total, 1 µg of RNA was reversely transcripted into complementary DNA using the iScript cDNA Synthesis Kit (Bio-Rad). qRT-PCR was performed using KAPA SYBR FAST qPCR Master Mix (2X) ABI Prism Kit (Kapa Biosystems). Glyceraldehyde 3-phosphate dehydrogenase (GAPDH) was used as the internal control. The conditions for the qRT-PCR reaction using the StepOnePlus Real-Time PCR System (Applied Biosystem) were 95°C for 5 minutes, followed by 40 cycles of 95°C for 30 seconds, and 61°C for 1 minute for GAPDH, while 59°C for 1 minute was used for Axin2 and DSPP. The forward and reverse primer sequences (Integrated DNA Technologies) for qRT-PCR (GAPDH, Axin2, and DSPP) were confirmed by BLASTN searches and are listed in Table 2 . The qRT-PCR was analyzed, and the data were calculated as 2 ^−ΔΔCt^ values, which were expressed relative to the control.

**Table 2:** Primer sequences used for qRT-PCR

Gene	Primer sequence	Temperature (°C)
Axin2	Forward: 5′-CCCCAAAGCAGCGGTGC-3′	59
	Reverse: 5′-GCGTGGACACCTGCCAG-3′	
DSPP	Forward: 5′-CTGTTGGGAAGAGCCAAGATAAG-3′	59
	Reverse: 5′-CCAAGATCATTCCATGTTGTCCT-3′	
GAPDH	Forward: 5′-GTCAGTGGTGGACCTGACCT-3′	61
	Reverse: 5′-AGGGGAGATTCAGTGTGGTG-3′	

### Ex-vivo Human Tooth Culture Model

The tooth samples were gathered after approval by the Medical Ethics Committee (COA. No. MU-DT/PY-IRB 2019/044.0807). The preparation of the human tooth culture model was performed according to the procedure of previous studies. 17 23 Immature human third molars from healthy young patients (15–18 years) with open apices and root lengths shorter than the crown length that was confirmed by pre-operative radiography were collected immediately upon extraction. The teeth were placed in 15 mL centrifuge tubes (Thermo Fisher Scientific) containing DMEM supplement with 300 IU/mL penicillin, 300 µg/mL streptomycin, and 0.75 µg/mL amphotericin B (Gibco, Life Technologies). The teeth were cleaned and sterilized with 0.12% chlorhexidine solution (CHX) for 10 seconds, followed by phosphate-buffered solution (PBS) for 30 seconds. The periodontal ligament and the remaining dental sac were removed with a sterilized No. 15 surgical blade (Swann-Morton Limited).

The teeth were handled using a sterile gauze soaked with the tooth culture medium. A Class I cavity was prepared until nearly exposing the pulp with a sterile diamond bur (ISO: 806 314 001 524 010, JOTA) at high-speed under sterile saline irrigation using a high-speed dental handpiece (Twinpower Turbine, J Morita). The pulp cavity was then intentionally exposed with a round carbide bur (ISO: 500 204 001 001 010, JOTA) at low speed without irrigation using a low-speed dental handpiece (US121, J Morita). Once exposed, the cavity was cleaned with sterile saline and finally dried with sterile cotton pellets.

**Fig. 1:**
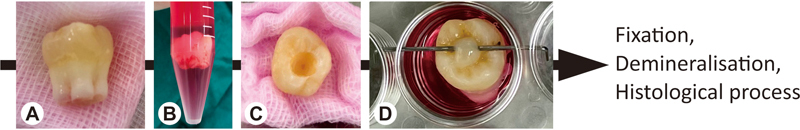
The tooth culture model set **-** up of the study. ( **A** ) Sample immature human third molar with one-to two **-** thirds root formation; ( **B** ) tooth transported in DMEM; ( **C** ) preparation of Class I cavity and pulpal exposure site; ( **D** ) fixation of the crown to a metallic wire with sealant and immediately suspended in culture medium.

The pulpal exposure site was lined with the experimental pulp capping materials, which were 45S5.1Li, 45S5.5Li, 45S5.1Zn, 45S5.5Zn, 45S5.1Zn sol-gel, 45S5.5Zn sol-gel, each in combination with Fibrinogen-Thrombin at 50 wt% concentration. Biodentine and a separate Fibrinogen-Thrombin mixture without additives were used as controls. After pulp capping, the cavity was bulk-filled with glass-ionomer cement (Equia Forte, GC). The occlusal crown was fixed to a metallic wire with a sealant (Clinpro Sealant, 3M ESPE) that was light-cured for 20 seconds (Bluephase N, Ivoclar-Vivadent) on 'high' mode (indicating an irradiance output of ∼1,100 mW/cm ^2^ ). The teeth were immediately suspended using the wire in separate wells of a 24-well culture plate (Nunc, Thermo Scientific) without allowing the root apices to contact the bottom of the wells. Each well contained a mixture of 2 mL DMEM supplemented with 10% FBS and an antibiotic–antimycotic. The culture medium was kept at 37°C in 5% CO _2_ and was refreshed daily over a 2- and 4-week culture period ( *n* = 3). The *ex-vivo* human tooth culture model set-up is presented in Fig. 1 .

### Histology and Immunohistochemistry

At the end of the culture period, the teeth were fixed with 4% formaldehyde (Formaldehyde-Solution, AppliChem) at 4°C for at least 3 days. The tooth enamel was removed with a high-speed diamond bur before being demineralized in 25 mL of 20% formic acid in a 50 mL centrifuge tube (Thermo Fisher Scientific) placed on a shaker (SSM4 Mini See-Saw Rocker). The acid solution was changed once a week. The endpoint of decalcification was determined by radiographs when complete radiolucency was observed. Once demineralized, the teeth were dehydrated through a series of ethanol dilutions and xylene before being embedded in paraffin. The teeth were longitudinally sliced at 5 µm thickness using a microtome (Leica RM2255). The tooth sections were mounted onto one glass slide for hematoxylin and eosin (H&E) staining and a separate positively charged glass slide for immunohistochemistry testing. H&E staining was done to evaluate the mineralization outcomes, where the slide was analyzed at 3 fields per sample for each culture period according to the following criteria: no foci, moderate foci (≤ 20 foci), and high foci (≥ 20 foci).

Immunohistochemistry was performed to distinguish the expression of DSPP, which is a molecular marker of odontoblasts that differentiate from dental pulp stem cells. The sectioned specimens on the positively charged slides were deparaffinized in xylene and rehydrated by placing in a serial dilution of ethanol, followed by antigen retrieval by heat-treatment in a 10 mmol citrate buffer at pH 6.0 (Dako Target Retrieval Solution 10X Concentrate) at 90°C for 10 minutes. The sectioned specimens were rinsed in PBS, then treated with 0.3% H _2_ O _2_ in methanol for 30 minutes to block the action of endogenous peroxidase. Afterward, the sectioned specimens were incubated overnight at 4°C in a moist chamber with primary DSPP antibodies (Dentin sialophosphoprotein DSPP Antibody LFMb-21, Santa Cruz Biotechnology) diluted in antibody–dilution buffer (Dako Antibody Diluent) at a 1:50 ratio. The sections were further treated with IHC HRP/DAB (Merck Millipore) according to the manufacturer's instructions. Finally, the sectioned specimens were counterstained with hematoxylin, dehydrated, and mounted with a glass slide cover (Menzel-Gläser, Thermo Scientific) before being observed under a light microscope (Olympus BX43, Olympus).

### Data Analysis

Statistical analyses were performed using Statistical Package for Social Sciences version 25.0 (IBM) at a 95% confidence level. Data distribution and homogeneity of variance were analyzed using Shapiro–Wilk tests and Levene's tests, respectively. The MTT data were analyzed using one-way ANOVA, and the different concentrations were compared using independent *t-* tests. The *Axin2* expression, DSPP expression, and mineralization outcome on the tooth culture model data were analyzed using Kruskal–Wallis H tests. The comparison between the control and experimental groups was done using Mann–Whitney *U* tests.

## Results

**Fig. 2:**
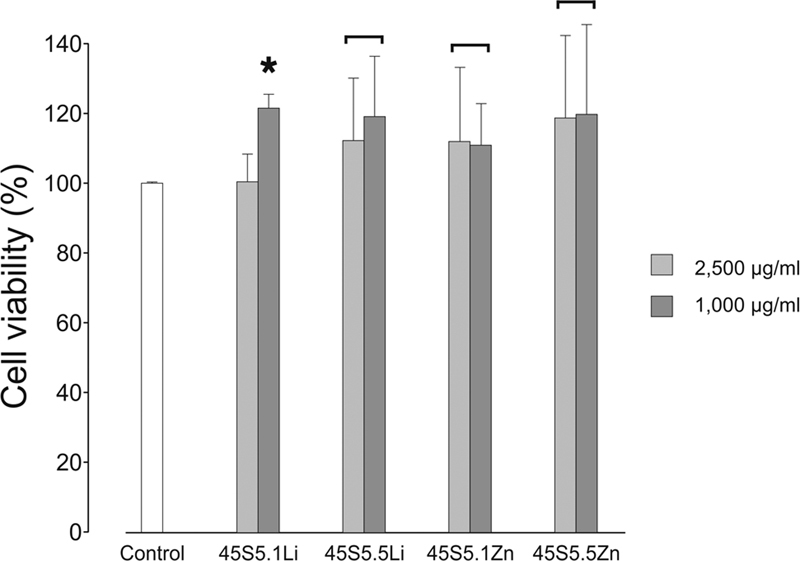
Percentage of cell viability for different compositions and concentrations of bioactive glasses. The connecting line between the different concentrations indicates no statistically significant differences **(** *p* > 0.05 **).** The asterisk denotes a statistically significant difference between the experimental group and the control **(** *p* < 0.05 **).**

The cytotoxicity of the experimental bioactive glasses is presented in Fig. 2 . The only statistically significant result was seen for the 1000 μg/mL concentration of 45S5.1Li, which had higher cell viability than the 2500 μg/mL concentration of the same composition as well as the control group. Other experimental groups did not show significant differences in cell viability with the control or between different concentrations of the same material.

The mean *Axin2* gene expression of the experimental groups over 24 hours is shown in Table 3 . The highest *Axin2* gene expression was detected at 12 hours, in which all experimental materials were significantly higher than Fibrinogen-Thrombin and the culture medium. However, the *Axin2* expression within each experimental group was not significantly different between the time periods of 30 minutes, 1 hour, 12 hours, and 24 hours.

**Table 3:** Mean ± SD (median) values of relative *Axin2* gene expression at 30 minutes, 1 hour, 12 hours, and 24 hours

Groups	30 minutes	1 hour	12 hours	24 hours	*p-* Value
45S5.1Li	2.6 ± 1.7	4.7 ± 1.0	5.9 ± 2.1	2.5 ± 1.2	0.079
	(3.0)	(4.9)	(6.4)*	(2.9)	
45S5.5Li	3.0 ± 2.0	4.9 ± 1.4	7.1 ± 1.0	3.5 ± 2.1	0.101
	(3.1)	(4.6)	(6.7)*	(4.5)	
45S5.1Zn	3.3 ± 1.5	4.1 ± 3.2	5.9 ± 2.1	3.0 ± 1.1	0.309
	(3.6)	(3.8)	(5.4)*	(3.0)	
45S5.5Zn	2.7 ± 1.3	3.4 ± 1.7	7.3 ± 1.9	3.6 ± 1.7	0.077
	(2.7)	(3.4)	(7.9)*	(4.5)	
45S5.1Zn sol-gel	3.0 ± 0.7	4.8 ± 3.7	7.7 ± 1.3	3.8 ± 2.0	0.168
	(3.3)	(4.5)	(7.4)*	(4.8)	
45S5.5Zn sol-gel	4.0 ± 2.7	4.4 ± 2.4	7.9 ± 1.6	4.8 ± 2.9	0.264
	(4.4)	(3.6)	(8.1)*	(5.7)	
Fibrinogen-Thrombin	1.3 ± 0.4	1.3 ± 0.3	1.5 ± 0.2	1.3 ± 0.2	0.788
	(1.2)	(1.4)	(1.6)	(1.2)	
Biodentine	4.8 ± 2.4	5.9 ± 3.2	9.4 ± 5.9	4.4 ± 0.8	0.319
	(5.1)	(5.1)	(6.2)*	(4.6)	
Differentiation medium	3.7 ± 2.1	3.7 ± 0.7	5.4 ± 2.4	3.8 ± 1.2	0.776
	(3.7)	(3.7)	(6.0)*	(3.4)	
Culture medium	1.4 ± 0.3	1.1 ± 0.3	1.1 ± 0.3	1.2 ± 0.9	0.690
	(1.3)	(1.2)	(1.3)	(1.1)	
*p* -Value	0.331	0.162	0.048*	0.164	

Note No significant differences among all groups at 30 minutes, 1 hour, and 24 hours ( *p* > 0.05).

* Significant differences between experimental groups and culture medium control group and Fibrinogen-Thrombin group ( *p* = 0.048) at 12 hour by Kruskal–Wallis test.

Regarding DSPP expression, the highest levels were seen on day 14 ( Table 4 ), in which all experimental groups were significantly higher than Fibrinogen-Thrombin and the culture medium. However, only the 45S5.1Zn sol-gel and 45S5.5Zn sol-gel groups had statistically significant elevated DSPP expression between the different time periods of day 3, 7, and 14.

**Table 4:** Mean ± SD (median) values of relative *DSPP* gene expression at days 3, 7, and 14

Groups	Day 3	Day 7	Day 14	*p-* Value
45S5.1Li	2.5 ± 0.5	5.1 ± 3.4	7.0 ± 2.4	0.113
	(2.2)	(4.3)	(6.9)*	
45S5.5Li	1.6 ± 0.7	4.9 ± 3.0	7.6 ± 1.5	0.079
	(1.2)	(4.2)	(7.5)*	
45S5.1Zn	2.6 ± 1.5	4.9 ± 2.1	7.6 ± 1.2	0.079
	(2.6)	(4.6)	(7.0)*	
45S5.5Zn	2.9 ± 1.9	5.2 ± 3.2	8.5 ± 1.4	0.113
	(1.9)	(5.5)	(8.2)*	
45S5.1Zn sol-gel	2.3 ± 0.1	4.9 ± 0.5	9.3 ± 0.8	0.027 #
	(2.2)	(4.9 # )	(9.1)* #	
45S5.5Zn sol-gel	2.6 ± 0.3	5.7 ± 2.2	9.9 ± 1.2	0.027 #
	(2.4)	(4.5 # )	(9.2)* #	
Fibrinogen-Thrombin	1.6 ± 0.3	1.5 ± 0.4	2.3 ± 0.4	0.05
	(1.5)	(1.3)	(2.1)	
Biodentine	1.5 ± 0.2	4.2 ± 3.8	8.3 ± 4.0	0.058
	(1.5)	(2.4)	(10.1)*	
Differentiation medium	3.5 ± 2.5	4.6 ± 2.2	8.5 ± 2.4	0.161
	(2.5)	(4.3)	(8.8)*	
Culture medium	1.6 ± 0.5	1.4 ± 0.4	2.1 ± 0.4	0.146
	(1.3)	(1.3)	(2.4)	
*p* -Value	0.183	0.126	0.040*	

Note: No significant differences among groups at day 3 and day 7 ( *p* > 0.05).

* Significant differences between experimental groups and culture medium control group, Fibrinogen-Thrombin group ( *p* = 0.040) at day 14 by Kruskal–Wallis test.

# Significant differences within material between day 3, 7, and 14 for 45S5.1Zn sol **-** gel and 45S5.5Zn sol **-** gel ( *p* < 0.05) by Kruskal–Wallis test.

**Fig. 3:**
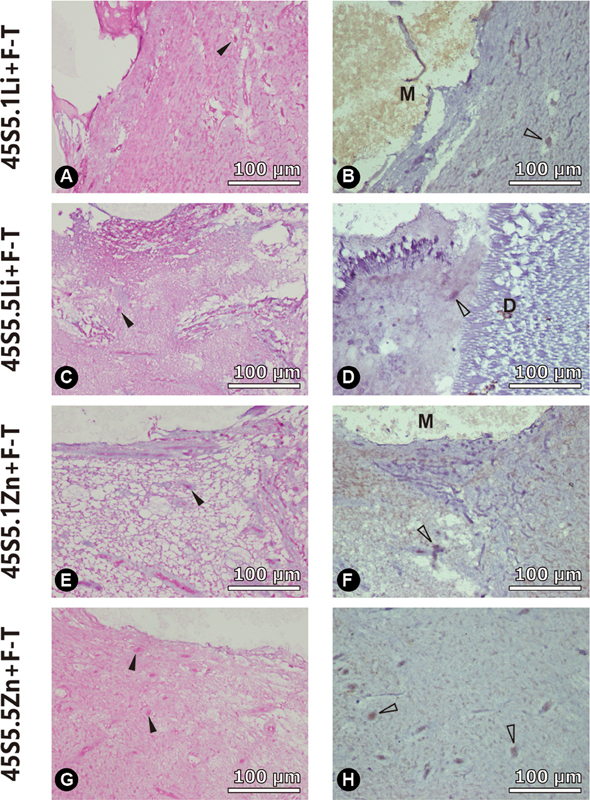
Representative histological sections of the tooth culture model at 4 weeks for different pulp capping materials as follows: Row (1) 45S5.1Li + F-T pulp capping material; Row (2) 45S5.5Li + F-T pulp capping material; Row (3) 45S5.1Zn + F-T pulp capping material; Row (4) 45S5.5Zn + F-T pulp capping material. ( **A, C, E, G** ) H&E staining at 40x magnification, dark arrows indicate mineralization foci; ( **B, D, F, H** ) Immunohistochemistry staining at 40x magnification, light arrows indicate DSPP expression from odontoblast-like cells. D: dentin; M: pulp capping material.

**Fig. 4:**
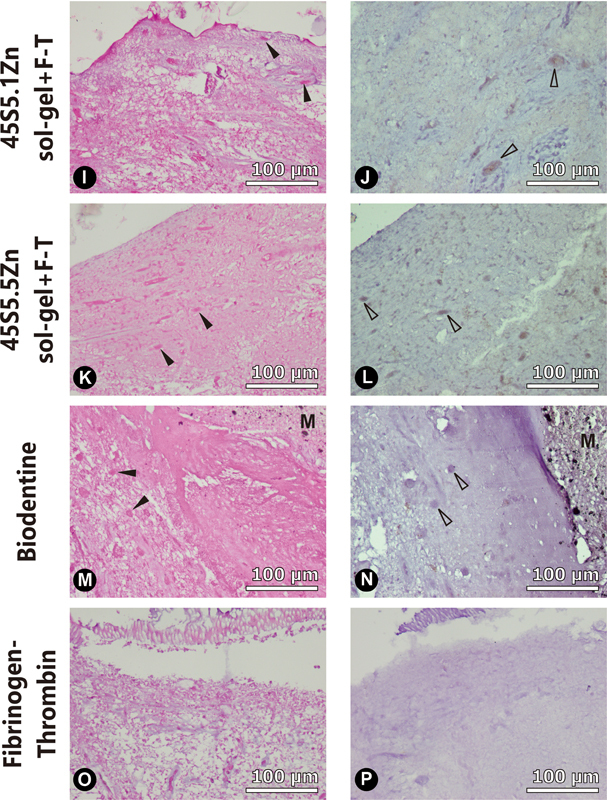
Representative histological sections of the tooth culture model at 4 weeks for different pulp capping materials as follows: Row (1) 45S5.1Zn sol-gel + F-T pulp capping material; Row (2) 45S5.5Zn sol-gel + F-T pulp capping material; Row (3) Biodentine; Row (4) Fibrinogen-Thrombin. ( **I, K, M, O** ) H&E staining at 40x magnification, dark arrows indicate mineralization foci; ( **J, L, N, P** ) Immunohistochemistry staining at 40x magnification, light arrows indicate DSPP expression from odontoblast-like cells. M: Pulp capping material.

Representative histological sections from the tooth culture model of each experimental group at 4 weeks are presented in Figs. 3 and 4 . Mineralization foci and odontoblast-like cells by H&E and immunohistochemistry staining, respectively, can be observed to be in close proximity to the pulp capping materials.

The evaluation of mineralization potential is presented in Table 5 . At 2 weeks, the presence of mineralization foci was not significant in all experimental materials, but at 4 weeks, the number of foci had increased significantly for the 45S5.5Zn + F-T, 45S5.1Zn sol-gel + F-T, 45S5.5Zn sol-gel + F-T, and Biodentine groups as compared with the Fibrinogen-Thrombin group.

**Table 5:** Evaluation of mineralization potential in tooth culture models at 2 and 4 weeks (number of specimens)

	45S5.1Li + F-T	45S5.5Li + F-T	45S5.1Zn + F-T	45S5.5Zn + F-T	45S5.1Zn sol-gel +F-T	45S5.5Zn sol-gel +F-T	Biodentine	Fibrinogen-Thrombin (F-T)	*p* -Value
*2 weeks*									0.363
None	2	2	2	1	1	0	1	3	
Moderate foci	1	1	1	2	2	3	2	0	
High foci	0	0	0	0	0	0	0	0	
*4 weeks*									0.031*
None foci	1	2	2	0	0	0	0	3	
Moderate foci	2	1	1	3	3	2	1	0	
High foci	0	0	0	0	0	1	2	0	
*p* -Value	0.114	0.317	0.317	0.025*	0.025*	0.034*	0.034*		

Note: No significant differences between groups at 2 weeks.

* Significant differences at 4 weeks between F-T group with 45S5.5Zn + F-T, 45S5.1Zn sol-gel + F-T, 45S5.5Zn sol-gel + F-T, and Biodentine groups.

## Discussion

This study evaluated the *Axin2* and *DSPP* expression in SHEDs in response to treatment with different experimental pulp capping materials. The first null hypothesis was rejected as the Li-and Zn-containing bioactive glass significantly increased the expression of *Axin2* and *DSPP* at 12 hours and 14 days, respectively. The second null hypothesis was also rejected as the mineralization foci in the tooth culture model were found to be significantly higher in the 45S5.5Zn + F-T, 45S5.1Zn sol-gel + F-T, 45S5.5Zn sol-gel + F-T, and Biodentine groups after 4 weeks.

The experimental bioactive glasses at 2,500 and 1,000 µg/mL concentrations can be considered non-cytotoxic as they demonstrated cell viability not only higher than the standard threshold of 70%, 24 25 but also higher than the control ( Fig. 2 ). Our results are related to those of previous studies which have suggested that the concentration of bioactive glass powder affects cell viability, where higher concentrations would induce more pH changes and release more ions to be more toxic to cells. 26 27 28 The non-cytotoxic concentration of Lithium-containing bioactive glasses has been reported to range from 750 µg/mL 28 to 6,000 µg/mL, 27 while for Zinc-containing bioactive glasses was from 50 µg/mL 29 to 1,500 µg/mL. 30 As the two concentrations in our study were within the threshold for cell viability, the higher concentration of 2,500 µg/mL was selected for further testing to utilize the higher ion release from the bioactive glass for studying *Axin2* and *DSPP* gene expression.

The *ex-vivo* tooth culture model has been used for studying the influence of capping materials on the early stages of pulp-dentine complex regeneration and mineralization. 6 17 21 23 As this model can be useful in evaluating the initial pulp response as well as biocompatibility of newly developed pulp capping agents, it would be able to represent and predict the initial reaction of pulp tissues to different capping materials in the clinical situation.

The Wnt/β-catenin signaling pathway is the basis for investigating the *Axin2* gene expression, which is also closely related to *DSPP* gene expression. 12 Although the Wnt/β-catenin signaling is not primarily responsible for dentine regeneration, the exogenous elevation of Wnt/β-catenin signaling can enhance tertiary dentine formation. 14

Li- and Zn-containing bioactive glasses have been purported to exhibit GSK-3 inhibition, which activates the Wnt/β-catenin pathway, leading to *Axin2* expression that regulates the differentiation of the odontoblast-like cells. The DSPP gene in these cells in turn encode for DSPP that is associated with the initiation of mineralization foci of the dentine matrix. 9 11 28 Besides Li and Zn, bioactive glasses that release calcium and silicon ions have also demonstrated the potential to induce human dental pulp stem cells to proliferate and differentiate into odontoblast-like cells. 9 11

In our study, *Axin2* gene expression at 12 hours from all experimental bioactive glasses was significantly higher than the control. Several other authors have reported similar observations, 27 28 although Neves et al found increased *Axin2* expression in mouse dental pulp cells as early as 1 hour after exposure to pulp capping materials. 12 Differences in cell source could explain the differences in cellular response rate i.e., the biological reactions in rats occur faster than in humans. Indeed, while dentin bridges were formed within 2 weeks to 1 month in experimental rat models, 13 dentin bridge formation in human dental pulp has been observed after ∼6 weeks to 2 months depending on the materials used and the study design. 31 Hence, the results of any study should be carefully interpreted to determine whether they would accurately reflect the conditions in living human subjects.

Bioactive glasses are commonly synthesized from two techniques, namely melt-quenching and sol–gel processes. 7 32 Each method produces different structural and mechanical properties, which influence the bioactivity of the materials. Conventional bioactive glasses are made by melt-quenching, producing a dense structure. In contrast, the sol–gel process results in more natural nano-porosities, resulting in high dissolution rates. 7 32

In the present study, the Li- and Zn-containing bioactive glasses produced by melt-quenching exhibited significantly greater *Axin2* and *DSPP* gene expression as well as mineralization compared with the control. Moreover, the most effective mineralization at 4 weeks for the melt-quenched bioactive glasses was shown by the group with the higher concentration of Zn (45S5.5Zn) ( Table 5 ).

Nevertheless, the results from the sol-gel bioactive glasses generally appeared to be superior to those of the melt-quenched materials. For instance, the 45S5.1Zn sol-gel and 45S5.5Zn sol-gel materials showed higher increments of DSPP expression over time. Although the DSPP gene levels were highest on Day 14 for all experimental materials, only these two sol–gel groups showed significantly different DSPP gene levels over the time periods ( Table 4 ). These results are consistent with several previous studies that showed strong upregulation of DSPP expression from human dental pulp stem cells exposed to bioactive glasses. 9 11 33

Besides, the sol–gel Zn-containing bioactive glasses displayed significant mineralization of the tooth model at 4 weeks compared with the Zn-containing bioactive glasses produced by melt-quenching ( Table 5 ). These findings could be related to the higher dissolution rate of the sol–gel bioactive glasses, which in turn results in enhanced bioactivity and biocompatibility. 7 32

Fibrinogen-Thrombin was chosen as a scaffold for the bioactive glasses to be used as pulp capping materials. Fibrinogen-Thrombin is a natural biomaterial that is biocompatible and biodegradable. 34 The gel form consists of a native matrix structure that is conducive to cell proliferation and angiogenesis. 35 36 It has also been proposed to be a more effective scaffold for tissue engineering, drug delivery, and cell delivery. 34

In the present study, both Fibrinogen-Thrombin and the control behaved similarly, where they did not influence either *Axin2* and *DSPP* gene expression or the formation of mineralization foci. The softness and biodegradability of Fibrinogen-Thrombin could potentially improve the biological properties of the biomaterial as it would allow a high concentration of ion release from the bioactive glasses. Therefore, the Fibrinogen-Thrombin gel can be recommended to be a suitable scaffold for pulp capping materials.

Biodentine demonstrated a promising mineralization potential as a mineralized nodule was observed next to the exposed area from the tooth culture model at 2 and 4 weeks, which is a comparable result to previous studies. 6 23 The CaCl _2_ in Biodentine increases the release of calcium ions which interact with phosphate from the tissue fluids to form calcium phosphate salts that undergo hydration to hydroxyapatite-like precipitate, promoting remineralization in the pulpal area in contact with Biodentine. 19 Moreover, Biodentine can induce dental pulp cells to secrete growth factor TGF- *β1* that in turn stimulates pulpal progenitor cells to migrate to the exposed site. 6 In addition, our results showed that Biodentine also influenced the Wnt signaling pathway as seen from the significant activation of *Axin2* expression at 12 hours and consequently of *DSPP* expression at day 14. These processes contributed to the formation of the highly mineralized nodule at both 2 and 4 weeks in our Biodentine samples.

## Conclusion

The tooth culture model is a valuable technique for studying initial pulp regeneration and mineralization. Bioactive glasses containing Lithium and Zinc significantly increased the *Axin2* and *DSPP* gene expression in SHEDs. Only Zn-containing bioactive glasses, particularly those produced by the sol–gel process, significantly enhanced DSPP expression and mineralization foci development in the tooth culture model. Therefore, Zn-containing bioactive glasses could be further developed as a potential pulp capping material.
